# Cardiovascular Stents: A Review of Past, Current, and Emerging Devices

**DOI:** 10.3390/ma14102498

**Published:** 2021-05-12

**Authors:** Alexandru Scafa Udriște, Adelina-Gabriela Niculescu, Alexandru Mihai Grumezescu, Elisabeta Bădilă

**Affiliations:** 1 Carol Davila University of Medicine and Pharmacy, 050474 Bucharest, Romania; alexscafa@yahoo.com (A.S.U.); elisabeta.badila@gmail.com (E.B.); 2 Cardiology Department, Clinical Emergency Hospital Bucharest, 014461 Bucharest, Romania; 3 Faculty of Engineering in Foreign Languages, University Politehnica of Bucharest, 060042 Bucharest, Romania; niculescu.adelina19@gmail.com; 4 Faculty of Applied Chemistry and Materials Science, University Politehnica of Bucharest, 060042 Bucharest, Romania; 5 Research Institute of the University of Bucharest—ICUB, University of Bucharest, 050657 Bucharest, Romania; 6 Internal Medicine Department, Clinical Emergency Hospital Bucharest, 014461 Bucharest, Romania

**Keywords:** cardiovascular stents, stent platform materials, stent optimization, surface functionalization

## Abstract

One of the leading causes of morbidity and mortality worldwide is coronary artery disease, a condition characterized by the narrowing of the artery due to plaque deposits. The standard of care for treating this disease is the introduction of a stent at the lesion site. This life-saving tubular device ensures vessel support, keeping the blood-flow path open so that the cardiac muscle receives its vital nutrients and oxygen supply. Several generations of stents have been iteratively developed towards improving patient outcomes and diminishing adverse side effects following the implanting procedure. Moving from bare-metal stents to drug-eluting stents, and recently reaching bioresorbable stents, this research field is under continuous development. To keep up with how stent technology has advanced in the past few decades, this paper reviews the evolution of these devices, focusing on how they can be further optimized towards creating an ideal vascular scaffold.

## 1. Introduction

Cardiovascular diseases have become an increasingly serious threat to human life; they are the leading cause of hospitalization and death globally [1,2,3,4,5]. Coronary artery disease (CAD), in particular, is the third most common cause of mortality worldwide, imposing a major health and economic burden on most developed nations [6,7,8,9,10,11]. CAD is characterized by the narrowing of the artery due to plaque deposits beneath the endothelium. Cells, fats, calcium, cellular debris, and other substances may accumulate in these deposits, starting a cascade of events—diminished blood vessel artery lumen, restricted blood flow, and inadequate nutrients and oxygen supply to the cardiac muscle—that can eventually cause myocardial infarction or transient cerebral ischemic attacks and stroke [12,13,14,15,16,17].

To restore normal blood flow and avoid the other critical consequences of vessel narrowing, special devices called stents can be inserted into the affected vessel using fluoroscopic and/or endoscopic guidance [13,14]. This procedure is minimally invasive compared to open cardiac surgery and is associated with lower mortality and morbidity in the long term and better outcomes in critically ill patients in the short term [15].

Therefore, cardiovascular stents are life-saving devices, rightfully included in the top ten medical breakthroughs of our century [18]. From a constructive point of view, stents are small, complex, cylindrically shaped hollow structures formed into a sequential ring construction comprising a series of struts and connecting elements [13,15,19]. The way that stents work relies on their design, which helps keep the path of human arteries through the body open [20,21].

Initially, bare-metal stents were used as a bail-out intervention scenario in the case of abrupt and threatened vessel closure with plain old balloon angioplasty. However, the reported positive outcomes encouraged stent placement to be adopted as the standard of care in percutaneous coronary intervention (PCI) [22]. Regardless of the history of clinical safety and efficacy, the usage of the first generation of these devices was frequently limited by in-stent restenosis, resulting in the failure of the existing stent or a reintervention using another stent [20].

Years later, as a solution to these issues, the deployment of drug-eluting stents (DESs) became an integral treatment option for patients with coronary artery disease [23]. Coronary DESs not only revolutionized PCI treatment but have also been approved as the current standard as they significantly reduce restenosis of vascular stents and mitigate the need for repeat revascularizations [20,24,25]. 

The decades of research and clinical trials in this field have resulted in various stent designs involving diverse materials, from which doctors can choose the appropriate device depending on the local deposition of plaque and fatty substances and the potential side effects [15,18]. 

This paper thus aims to review past and currently available stents and shed some light on how these devices can be further optimized towards ideal in vivo behavior.

## 2. Evolution of Cardiovascular Stents 

**Figure 1:**
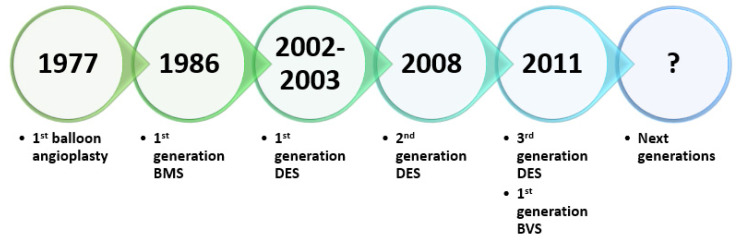
Cardiovascular stents evolution—a brief timeline. Created based on information from the literature [7,27,28,29].

The rapid technological advancements that have taken place over the past 40 years have greatly affected PCI evolution [26]. Interventional cardiology has undoubtedly evolved since the first percutaneous transluminal coronary angioplasty. This evolution began with a balloon catheter mounted on a fixed wire and progressed into bare-metal stents (BMSs), first-generation DESs, and second- and third-generation biodegradable polymer-based DESs, culminating in the introduction of bioresorbable vascular scaffolds (BVSs), which are currently under development [7,17] (Figure 1).

**Figure 2:**
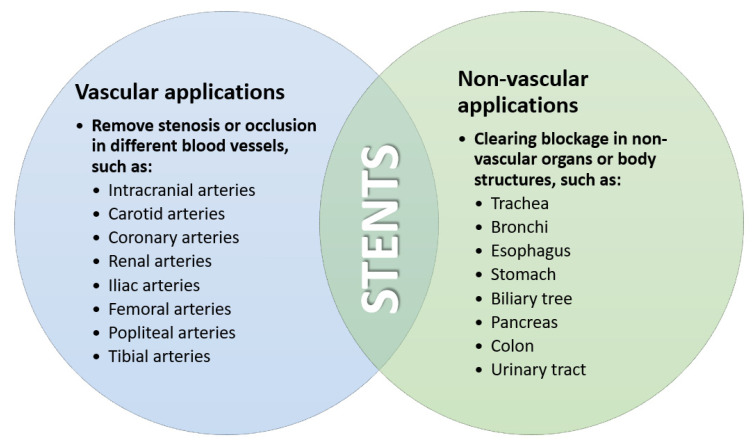
Medical applications of stents. Created based on information from [20].

Stents s were found to have versatile medical applications (Figure 2) in a wide variety of clinical emergencies after their introduction by Charles Theodore Dotter in the 1960s in the US, but their potential utilization in CAD had not yet been discovered [20,29]. In the late 1970s, two intervention techniques were introduced: coronary angiography and balloon angioplasty. In the beginning, plain balloon angioplasty without stent implantation showed high rates of arterial recoil and vessel dissection [6].

### 2.1. Bare-Metal Stents

The first coronary stent was implanted in 1986 and became the inception point for the first generation of BMSs [6,7]. These devices were usually fabricated from corrosion-resistant materials, such as stainless steel (316L), cobalt-chromium (Co-Cr), platinum-iridium (Pt-Ir) alloys, tantalum (Ta) or nitinol (Ni-Ti), and were permanently implanted [30,31,32,33]. 

A common challenge faced when designing stents is the recoil phenomenon, which refers to the percent decrease of stent diameter between its expanded and relaxed forms [13]. To avoid significant changes in their dimensions, stent materials must have adequate mechanical properties (Table 1). A common manufacturing technique for all materials is laser cutting, but nitinol has also been processed into stents using thin-film technology followed by a photoetching step [34,35,36].

The above-listed materials have suitable properties for providing the lesion site’s required support while preserving device shape and integrity, but their non-degradability leads to a series of post-intervention complications [32]. Some of the recorded issues include: (sub)acute occlusion and neointimal hyperplasia resulting in the in-stent recurrence of stenosis (in-stent restenosis) due to both arterial damage and stent implantation, late-stage thrombosis, chronic inflammatory response, and the necessity of keeping the stent as a foreign body throughout the entire life [31,32,43,44]. 

Stainless steel is a resistant, hard, non-corrosive material, yet its limited biocompatibility represents a major drawback in terms of thrombosis formation. On the other hand, nitinol alloy has better biocompatibility in the short term but, in time, nickel can migrate from the material and produce immune response issues. Tantalum has very good mechanical properties and it is biologically inert. This metal lacks ferromagnetism, is very stable and resistant, and its oxide surface layer developed after implantation is biocompatible. However, no difference has been noticed in the thrombosis rate when comparing this material with stainless steel [14].

To prevent the cascade of life-threatening events, another generation of stents had to be developed.

### 2.2. Drug-Eluting Stents

**Figure 3:**
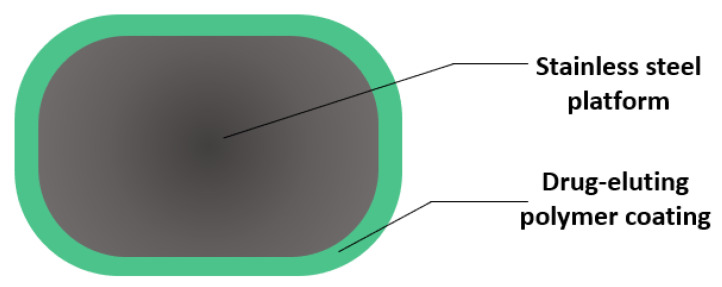
Cross-section view of a drug-eluting stent strut. Created based on information from the literature [50,51].

An important turning point for interventional cardiology was reached in 2002 when the first DESs were introduced into the market. The structure of the first generation of DESs was based on a stainless-steel platform coated with a drug-eluting durable polymer [6,23,45] (Figure 3). Such devices allow localized elution over the course of a month of neointimal inhibiting drugs, such as sirolimus and paclitaxel, which have antiproliferative effects [46,47,48,49].

DESs were mainly adopted as a solution for in-stent restenosis, a major problem associated with the stenting procedure [52]. Their proven long-term safety and efficacy are also reflected in the improved subsequent revascularization rates and reduced risk of thrombosis compared to BMSs [53,54,55,56]. 

Nonetheless, the first generation of DESs required longer dual antiplatelet therapy (DAPT) than BMSs, which can be accompanied by an increase in bleeding [55,56,57]. Specifically, the risk of stent thrombosis with BMSs is the highest within the first 14 to 30 days; hence, DAPT is routinely recommended for at least one month, while a period of at least 6 to 12 months is necessary after DES implantation to avoid late stent thrombosis [58,59,60]. Moreover, randomized and observational studies have reported a steady increase in the cumulative incidence of late and very late stent thrombosis. In contrast, pathogenic studies have shown delayed arterial healing [61,62], mainly due to the long-term inflammation of the peri-stent tissue caused by the durable polymers used as drug carriers [45].

To overcome these limitations, the second generation of DESs was developed starting from 2008. These new devices presented improvements in strut thickness, deliverability, and flexibility. [6]. Efforts were made to change all three components (platform, coating, and drug) of previous-generation stents [49]. Other materials were proposed for the metallic platform, namely cobalt-chromium and platinum-chromium alloys, as they allowed a reduction in the strut thickness [6]. The extra benefits that the new platform alloys brought were superior radial force and better radiopacity, with thinner strut formulations than the 316L stainless steel used in previous stent generations [12,23,63]. 

Another important component of DESs is the stent coating, for which biodegradable polymers have been considered as drug-eluting layers [45]. The coating must provide structural integrity, consistent dosing, and controlled release kinetics, as it serves as the interface between the stent and the vascular tissue. Thus, the biocompatibility of the polymers used for coating stents is of vital importance. As the polymer coating also acts as the eluting drug’s reservoir, it must be non-thrombogenic, non-inflammatory, non-toxic to cells, and should encourage arterial healing by re-endothelialization [12,19,21,23,63]. In this respect, the adequate features of phosphorylcholine, polylactic acid, poly(vinylidencefluoride-co-hexafluoropropenen), and polyvinylpyrrolidone have attracted interest for stent coatings, mitigating inflammation and thrombosis risk [6,24,64]. Furthermore, there are many available techniques for fabricating polymer-coating stents that can be chosen from (e.g., dip-coating, electrospinning, spray coating, hot melt coating), depending upon the constitutional complexity of the stent platforms and their uncharacterized properties as medical devices [20,65,66,67].

Regarding the incorporated drugs, new substances have been proposed in these stents, such as zotarolimus, everolimus, and novolimus [6,68,69,70,71,72]. Through their diffusion into the vessel wall, these compounds lead to good endothelial coverage, inhibition of vascular restenosis, and suppression of transplant rejection [20,22,52,63]. Moreover, drug diffusivity is highly dependent on the size and charge of the drug. Specifically, high molecular weight anionic substances have a lower diffusivity than neutral or cationic low molecular weight compounds [73,74]. Hence, these characteristics must be considered when choosing which drug is to be included in the coating.

However, the long-term durability of DESs is still not optimal, posing limitations in terms of adaptive remodeling due to vessel caging by metal prosthesis, abnormal coronary vasomotion, and undefined interactions of antiproliferative drugs [75,76,77]. Therefore, more recent research has shifted towards developing non-permanent devices.

### 2.3. Bioresorbable Stents

Generally, stents are needed temporarily (until healing and re-endothelialization are obtained) for their short-term benefits, while in the long term they tend to create severe complications associated with leftover metal. To diminish adverse effects, like chronic inflammation, restenosis, late-stage thrombosis, and vessel size mismatch, a new generation of devices (described in the literature as bioresorbable stents, biodegradable stents, or bioresorbable vascular scaffolds) is currently being developed [25,30,32,75,78,79,80,81]. 

The use of bioresorbable stents is considered a revolution in interventional cardiology [82]. Such devices are fabricated from materials that provide transient support and progressively degrade, being dissolved or absorbed in the body after the remodeling process [13,30,31,79,83]. It is in this regard that biocompatible, biodegradable polymers and metallic materials have attracted increased attention [32,84] (Table 2). Manufacturing methods include, but are not limited to, photochemical etching [85,86], helical coiling [87,88], braiding techniques [89,90,91], fluid dispensing [92], three-dimensional (3D) printing [93,94], electrospinning [95,96], hot extrusion [97], and selective laser melting [98]. 

**Figure 4:**
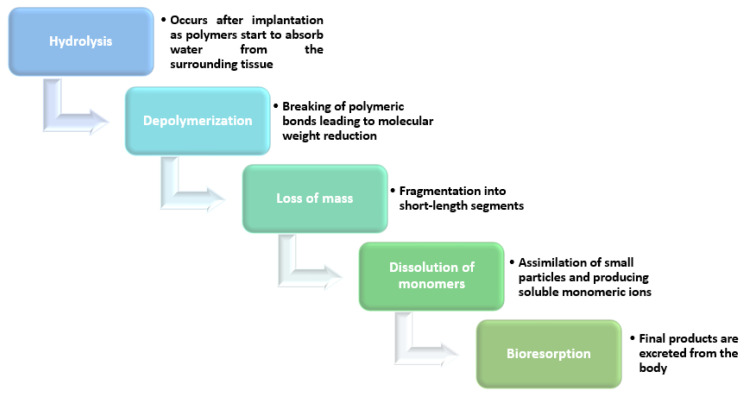
Disintegration steps of polymer-based bioresorbable stents. Created based on information from the literature [103,104,105,106].

Out of these materials, PLLA was used for the first European Medicines Agency-approved BVS [27] and is the most typical biocompatible polymer used for current bioresorbable stents [31,84]. PLA is another biodegradable polymer with satisfactory processing characteristics and mechanical properties and can be employed either alone or in polymer blends to fabricate new generation stents [102]. Other polymers that can be resorbed in the organism (Figure 4) within several months of stenting are PLGA, PGA, PCL, polyorthoesters, phosphorylcholine, fibrin, hyaluronic acid, and polyethylene oxide/polybutylene terephthalate [31].

**Figure 5:**
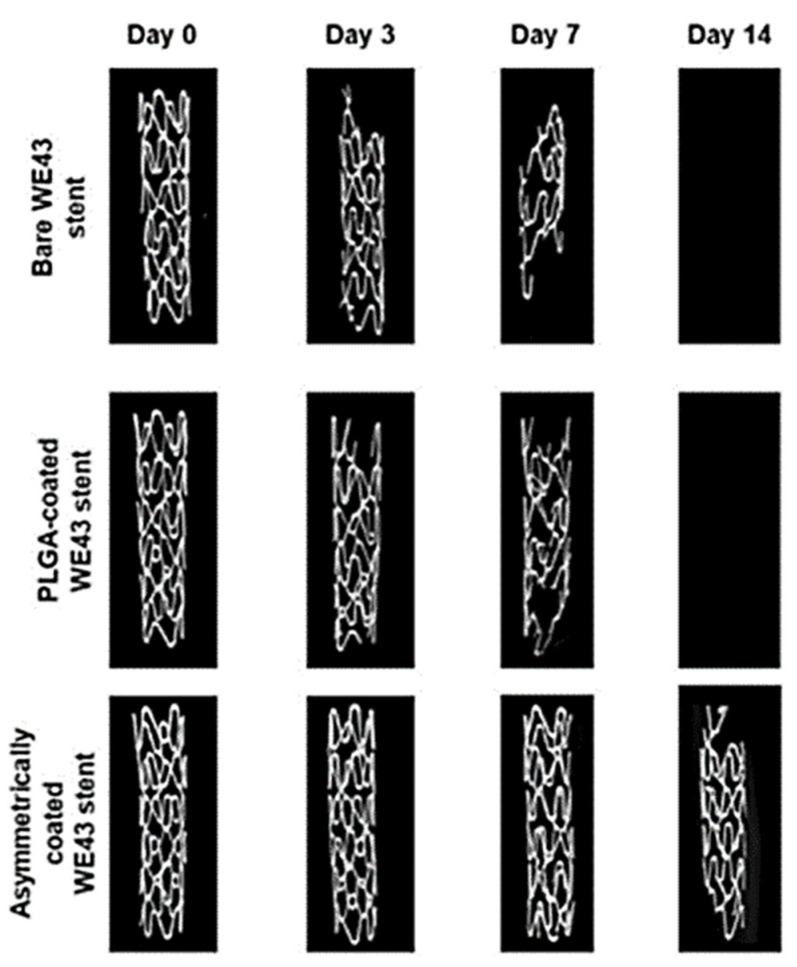
Disintegration of metal-based bioresorbable stents. Reprinted from an open-access source [109].

However, as can been seen in Table 2, biodegradable metals are endowed with better mechanical properties than polymeric materials. Biodegradable metallic stents are considered to be a revolutionary alternative to permanent stents [107]. Magnesium alloys, in particular, offer superior elastic moduli and tensile strengths while maintaining a uniform degradation over a similar or shorter time [108]. Another convenient property of magnesium (Mg) alloys is their electronegative charge during degradation, which provides antithrombotic potential [101]. However, bare Mg alloy stents tend to corrode too fast, requiring polymer coatings to slow down their degradation process and sustain the drug-delivery capability [109] (Figure 5). Other metal-based materials for new-generation stents are iron and zinc alloys. They have been proven to be well-tolerated in vivo, having similar mechanical properties to non-degradable metals [31,32,110].

By reducing the contact time between the stent surface and the blood flow, bioabsorbable stents provide positive outward remodeling after biodegradation, an improved possibility of later surgical revascularization, facilitation of secondary re-intervention, and a lower risk of late stent thrombosis [19,25,80,111].

Nonetheless, several side effects have also been reported from BVSs. Their recorded drawbacks include poor healing, platelet deposition, poor deliverability, rheological disturbances, and increased scaffold fracture risk [19]. Therefore, there is still room for improvement, and ongoing research must address all these issues before being able to provide an ideal stent. 

It is possible to create better solutions for CAD treatment in the future only by knowing and understanding the characteristics and outcomes of already available stents. In this respect, a summary of observations from clinical trials regarding several stent devices is presented in Table 3.

## 3. Stent Optimization

### 3.1. Features of an Ideal Stent

**Figure 6:**
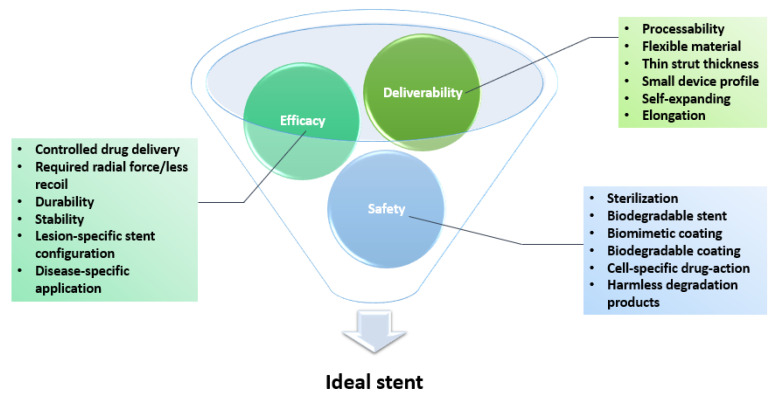
Schematic representation of an ideal stent’s properties. Created based on information from the literature [23,82,84,103,135].

For a stent to be considered ideal, it should exhibit good biocompatibility, flexibility, and deliverability, strong radial force, and good radiopacity under fluoroscopy. An ideal device should also result in low rates of thrombogenesis, neointimal hyperplasia, and stent thrombosis during long-term follow-up [6,128,129,130]. To provide effective treatment for CAD, the stent must not interact with the active restenotic drug, must release the drug at the proper rate, and must be biologically inert and mechanically stable over the long term. It should cause minimal trauma to the vessel wall, cause a minimal inflammatory reaction, reendothelialize well, provide scaffolding for the vessel, and finally promote vessel healing and remodeling [12,22,131,132,133,134]. The multitude of different and occasionally conflicting requirements presented above is separated in the literature into three main categories, each with several underlying criteria: deliverability, efficacy, and safety [23] (Figure 6).

To meet most, if not all, of these features, stent optimization can be obtained through several approaches, such as developing novel stent platforms (either by using new materials or creating new designs), functionalizing stent surfaces, and adopting more precise manufacturing technologies.

### 3.2. Novel Platforms

**Figure 7:**
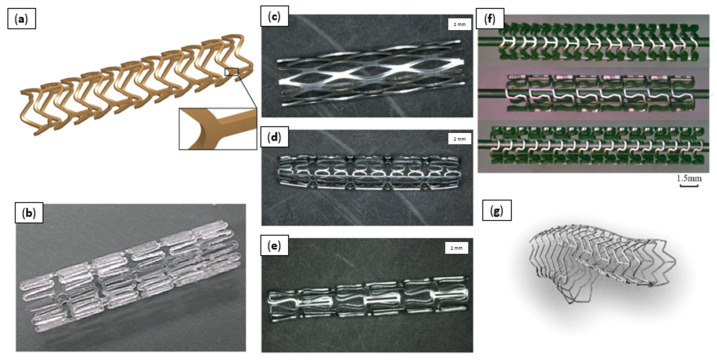
Various stent platforms. (**a**) PLGA bioresorbable polymer constitutive model [2]; (**b**) edge-rounded PLA biomedical stent [102]; (**c**) Zn stent with rhombus design [110]; (**d**) Zn stent with U-design [110]; (**e**) Zn stent with Omega design [110]; (**f**) Mg alloy AE21 stents [101]; (**g**) main vessel stent with side-branch scaffold [140]. Reprinted from open-access sources.

Various new stent platforms are under development or have even reached the clinical trials stage (Figure 7). Researchers have proven that stent geometry is a determining factor in restenosis [136]. Assuming that the material and surface area remain unchanged, an increase in the number of support struts causes a proportional increase in the neointimal area, reducing vascular damage [137]. 

As strut thickness should remain as low as possible to avoid restenosis, the strength cannot be increased by implanting bulkier devices. Therefore, one solution is to optimize the stent pattern so that the device’s mechanical performance is enhanced through the adjustment of strain distribution and evolution during stent deformation [101]. 

Another example of design improvement is the manufacture of bifurcation stents, which can overcome the challenges faced during bifurcation procedures [19,138]. PCI in bifurcations is associated with higher rates of periprocedural complications, in-stent restenosis, and stent thrombosis [139]. Dedicated bifurcation stents can be chosen for this procedure as they make it possible for the operator to perform the lesion stenting without the need to rewire the side branch [140]. This alternative to complex two-stent strategies has led to similar clinical outcomes as the conventional provisional stenting approach [141].

It is not only the stent design that can be improved; new materials are also being investigated. One metal in particular has attracted attention for these biomedical implants, and that is zinc [110]. Despite not yet being actively used for bioresorbable stents, zinc-based materials are considered better than magnesium alloys. They have an ideal rate of in vivo degradation (mechanical integrity maintained for 6 months and ~50% degradation 12 months after implantation), good overall biocompatibility, and result in less proliferation of smooth muscle cells and a good antibacterial effect [137,142,143]. Additionally, zinc is more ductile than magnesium, facilitating the processing of these alloys into the desired design. The range of mechanical properties attainable by Zn alloys cover the needs of cardiovascular stents, as these materials exhibit ultimate tensile strengths varying from 87 to 399 MPa and elongation-at-break values from 0.9% to ~170% [110]. Other platform materials tested for bioresorbable stents include iron alloys. As iron has a slow degradation rate, combining it with other metals in adequate proportions accelerates corrosion without sacrificing the required mechanical properties. Specifically, promising results have been obtained with Fe-35Mn (iron alloy containing ~35% manganese), which has similar ultimate tensile strength and yield strength to stainless steel but with a degradation rate that is three to eight times faster [82].

Despite the more frequent use of crystalline alloys, multi-component metallic alloys with disordered atomic distribution have recently become a topic of research for their potential improvements to stent performance [144,145,146,147,148]. These new materials, generally known as bulk metallic glasses (BMGs), present a unique combination of glassy structures and metallic bonds that places them at the frontier of biomaterials research [149,150]. Notably, Zr-based BMGs satisfy the ideal features of stents to a great extent, exhibiting excellent mechanical properties, high corrosion resistance, and good biocompatibility. Their enhanced hardness and strength compared to traditional materials allow for the design of markedly thinner stent struts, significantly diminishing restenosis risk and improving device deliverability [144,146,147,151,152,153]. In particular, BMGs are being investigated for use in self-expanding stent applications as their elastic spring-like restoration is significantly better than that of nitinol-based devices [150]. Nonetheless, these materials lack ductility and are susceptible to catastrophic failure at room temperature due to highly localized plastic deformation beyond their elastic limit [149,150]. Therefore, further in vitro and in vivo investigations are required before implementing BMGs in clinical practices [144].

Research has also focused on the shape memory effects of several materials, which can be highly advantageous in fabricating innovative stents. Shape memory behavior is especially useful for stent delivery, allowing for catheter size reduction and self-deployment without auxiliary devices. This effect occurs when the material undergoes a change in its crystal form and results in the material’s ability to recover an original shape in response to a stimulus [154,155,156]. Specifically, in the case of shape-memory alloys (e.g., nitinol), there is a reversible transformation from the austenite phase to the martensite phase, which takes place over a specific temperature range depending on the alloy composition [157,158,159]. Similarly, polymeric smart materials that recover from a deformed state to their original shape under external stimuli have also been considered [160,161,162]. Stents from shape-memory polymers (e.g., poly(tert-butyl acrylate) and poly(ethylene glycol) dimethylacrylate) could be manufactured to preserve shape storage at ambient temperature and become fully activated at body temperature [156]. Compared to shape-memory alloys, these polymers are considered better for stent applications in terms of recoverable strain, processability, cost effectiveness, and tunability of properties [157,163]. Besides temperature-triggered transformations, light-responsive and chemically responsive shape recoveries have been investigated as alternative mechanisms to reduce tissue damage from higher heat transitions [154,164,165,166,167].

Another innovative approach is to include the drug directly into the stent platform instead of using a polymer coating. Such devices are called drug-filled stents (DFSs) and provide controlled elution from an internal stent lumen, avoiding the inflammation associated with polymers from earlier generations of DESs [168,169]. Early trials were considered successful as DFSs presented promising results, such as minimal neointimal hyperplasia and a high degree of stent strut coverage at one month after implantation in optical coherence tomography [49,170]. 

Another interesting development is the application of an antiproliferative drug as a coating on the surface of a balloon instead of a regular stent platform. In this case, medication is delivered locally to the tissue using prolonged 60 s inflation. Drug-coated balloons are particularly attractive in treating *de novo* lesions, especially in small-vessel disease. This method has shown similar results in treating restenosis as implanting a second DES layer, but the much higher price limits its use [49,171,172,173]. 

### 3.3. Surface Modifications

A different approach for improving the biocompatibility, safety, and efficacy of stents is to modify the surface of the implant [174]. The biological response can be improved through surface changes in three categories: topographical modifications, physicochemical modifications, and surface biofunctionalization [80]. 

Topographical modifications refer to the creation of specific nano- or micro-patterned surfaces with the purpose of accelerating endothelial healing [80]. The idea behind this strategy is to obtain a controlled biomimetic profile that can increase the adhesion and migration of endothelial cells onto the stent surface. In this respect, the pattern’s depths must be in the sub-micron range to avoid unwanted platelet adhesion [175]. Various surface treatments can be applied to the stent platforms to obtain smooth, contamination-free surfaces, such as mechanical polishing, electropolishing, ultrasonic cleaning, chemical etching and degreasing, and low-pressure plasma etching [136]. 

Endothelial adhesion and spreading can also be modified through physicochemical changes. These can be performed by generating suitable functional groups and/or modulating surface energy [80]. For this purpose, oxides and nitrides of metals can be used. Alternatively, metals and polymers can be deposited through various physicochemical techniques, including magnetron sputtering, pulsed laser deposition, and matrix-assisted pulsed laser evaporation. Another example of chemical surface modification of stents is the molecular layer deposition of silanes, which are compounds rich in useful functional groups [31]. 

Another category of surface modifications is biofunctionalization, which refers to the surface immobilization of biomolecules with specific biological properties while the original mechanical properties of the material remain unchanged [76]. Cell–material interactions can be improved by using biomacromolecules (e.g., heparin, fucoidan, chondroitin sulfate, hyaluronic acid, antioxidant compounds, or collagen) that facilitate a cascade of events beneficial for regenerating the damaged area with a functional endothelium [80,174]. 

Surface heparinization is one of the most common histocompatibility-enhancing methods. This is due to heparin’s useful effects in preventing intimal hyperplasia and inhibiting smooth-muscle cell proliferation and migration. The negative charge of heparin, conferred by its many sulfo and carboxyl groups, mediates the interactions with enzymes, esterase inhibitors, protease, chemokines, and growth factors [76,176]. 

Another solution is the use of a CD31-mimetic peptide to favor vascular homeostasis and arterial wall healing. Such a surface functionalization has been proven to be successful: one week after implantation, CD31-mimetic struts were reportedly fully endothelialized with no activated platelets/leukocytes, while four weeks after stenting a significant reduction in neointima development was noticed compared to bare-metal stents [177]. 

The application of coatings also fits under the broad umbrella of surface modifications. The development of coating materials and designs that facilitate drug delivery while maintaining suitable biological properties is an intense field of research. Of particular interest are multifunctional coatings that synergistically combine features, such as modification of the bulk material’s degradation rate, reduction of the risk of thrombosis, acceleration of the proliferation of endothelial cells, or even endowment of the device with new functionalities. 

One such development is available for magnesium-based stents, which can be improved through alkali treatment followed by polydopamine and hyaluronic acid immobilization via strong electrostatic adsorption and covalent bonding between the carboxyl group of hyaluronic acid and the amine or hydroxyl groups of polydopamine. Hence, a magnesium/OH/polydopamine/hyaluronic acid coating can be obtained, the optimum biocompatibility–antithrombogenicity balance of which is achieved by adjusting the hyaluronic acid content on the polydopamine surface [178].

Another innovative approach is the inclusion of biomarkers in the stent coating. In this respect, researchers have proposed using CD146 as a distinctive target for selectively capturing endothelial progenitor cells. The authors of one study immobilized these antibodies on cobalt-chromium stents coated with silicone nanofilaments, resulting in superior devices that accelerate re-endothelialization and prevent artery restenosis [179]. 

The current guidance technique for PCI is X-ray fluoroscopy, which, due to its poor soft-tissue contrast and limitation to a single plane, hinders the precise navigation of endovascular instruments. Safer and more accurate guidance can be achieved through magnetic resonance imaging (MRI), but the availability of MRI-visible stents is limited. One way to solve this problem is through the creation of superparamagnetic iron oxide (SPIO)-functionalized devices that allow better control of stent deployment, subsequently reducing the rate of implantation-associated complications. Such stents can be obtained by incorporating SPIO nanoparticles in a biocompatible polymer (i.e., PLGA) that serves as a multifunctional coating [180].

## 4. Future Perspectives

Currently, stents are mostly produced by laser cutting or other fabrication techniques, like electrode discharge machining, waterjet cutting, photochemical etching from tubing, and various wire-forming techniques, such as braiding and knitting [136,137]. However, these technologies can be updated or replaced through advancements in augmented reality (AR), 3D printing, and deep learning (DL) [19]. Specifically, by making use of the information gathered about blood vessels through AR and DL, materials like PLA [156], polydiolcitrate [94], or metallic glasses [181] can be 3D printed into cardiovascular devices with more distinct designs than commercially available ones [19,182,183,184,185]. 

The use of such technologies opens the door for patient-specific devices that can meet each individual’s exact requirements. In this way, the challenges of immunogenicity, inflammation, fibrous tissue formation, material degradation, and cytotoxicity can be addressed by creating customized cardiovascular stents corresponding to the target blood vessels’ physiological conditions and pathological status [19]. 

Furthermore, smart stents can be introduced instead of simple-support devices. Researchers have proposed innovative implantable and biocompatible platforms that can measure blood flow using miniaturized ultrasonic transducers. Such systems offer flexibility as they can both transmit and receive information in a wireless manner. Therefore, smart stents would be able to prevent restenosis while simultaneously monitoring post-implantation outcomes on the spot [16]. 

Another future direction is the replacement of the traditional implantation procedure. Considering the success of drug delivery through blood vessels enabled by a high-precision, biocompatible, and 3D-printable micro-robot, the developers, from ETH Zurich, have started to investigate micro-robots for stent deployment. Moreover, at the same university, novel 4D printing technology has been introduced for the fabrication of cardiovascular stents with dimensions 40 times smaller than currently existing ones [13].

## 5. Conclusions

To summarize, cardiovascular diseases pose a severe threat to a large part of the global population, affecting both life quality and duration. Particularly, arterial stenosis caused by plaque deposition stands behind an aggravating cascade of events. 

Each step of the technological progress in intracoronary stents has influenced percutaneous coronary intervention by improving its outcomes in both the short and long terms. Drug-eluting stents have become a standard of care in PCI, as contemporary DES platforms incorporate significant advances in scaffold design, polymer compatibility, and antiproliferative drug delivery. Moreover, bioresorbable stents have recently emerged as a convenient solution where permanent stents are required, as their degradation make it possible to avoid undesired long-term effects. 

However, challenges can still arise and there are still individuals suffering from stenting failure, thrombosis, or restenosis, regardless of the excellent safety and efficacy of the newest devices compared to predecessor generations. Therefore, new materials for both stent platforms and polymer coatings must be investigated and better stent designs developed. 

To conclude, despite being used for decades, stents still have considerable research potential. Stent optimization should be achieved in all the involved stages, from the design, choice of materials, and fabrication method to the surface functionalization and implantation procedure. 

## Figures and Tables

**Table 1:** Mechanical properties of the most common stent metals and alloys.

Stent Material	Young’s Modulus (GPa)	Ultimate Tensile Strength (MPa)	Equivalent Von-Mises Stress (MPa)	Elongation at Break (%)	References
Iron	211	270	-	40	[33,37,38,39]
Stainless steel	193	595	231.14	40	[15,33]
Tantalum	186	285	514.70	-	[15,40]
Nitinol	45–50	1200	436.12	~20	[15,41]
Cobalt-chromium L-605	243	1020	536.20	50	[15,42]
Cobalt-chromium MP35 N	233	930	529.82	45	[15,42]

**Table 2:** Important properties of several biodegradable stent materials.

Stent Material	Young’s Modulus (GPa)	Tensile Strength (MPa)	Elongation at Break (%)	Degradation (Months)	References
PLA	2–4	65	2–6	18–30	[99,100]
PDLLA	1–3.5	40	1–2	3–4	[99]
PLLA	2–4	60–70	2–6	>24	[99]
PGA	6–7	90–110	1–2	4–6	[99]
PDLGA (50/50)	1–4.3	45	1–4	1–2	[99]
PLGA (82/12)	3.3–3.5	65	2–6	12–18	[99]
PCL	0.34–0.36	23	>4000	24–36	[99]
PLA/PCL (70/30)	0.02–0.04	2–4.5	>100	12–24	[99]
PC	2–2.4	55–75	80–150	>14	[99]
AE21	45	-	-	2–3	[78,101]
AE42	45	237	8–10	-	[78]
WE43	40–50	220–330	2–20	3–12	[99]
AZ31	45	235	7–21	<4	[78]

Abbreviations: PLA—polylactic acid; PDLLA—poly-DL-lactic acid; PLLA—poly-L-lactic acid; PGA—polyglycolide; PDLGA—poly-DL-lactide-co-glycolide; PLGA—poly-lactic-co-glycolide; PCL—polycaprolactone; PLA/PCL—polylactic acid/polycaprolactone; PC—polycarbonates; AE21—magnesium alloy containing ~2% aluminum and ~1% rare earth metals; AE42—magnesium alloy containing ~4% aluminum and ~2% rare earth metals; WE43—magnesium alloy containing 4.2% yttrium, 2.4% neodymium, 0.6% cerium/lanthanum, and 0.5% zirconium; AZ31—magnesium alloy containing ~3% aluminum and ~1% zinc.

**Table 3:** Characteristics of developed and under-development stents.

Device	Stent Specifications				Observations	References
	Platform Material	Strut Thickness (μm)	Coating Material	Drug		
Cypher	Stainless steel	140	Parylene C	Sirolimus	Drug-eluting time: 80% elutes in the first 30 days, while the remainder is released by the end of 90 days Outcomes at 1 year (percent from the total number of patients in the trial): - target vessel re-vascularization: 8.1% - stent thrombosis: 1.2% - cardiac mortality: 1.4%	[31,112,113]
Taxus	Stainless steel	132	Polystyrene-b-isobutylene-b-styrene (translute) polymer	Paclitaxel	Drug-eluting time: elutes over 90 days Outcomes at 1 year (percent from the total number of patients in the trial): - target vessel re-vascularization: 7% - stent thrombosis: 0.7% - cardiac mortality: 3.1%	[31,112,113]
Axxion	Stainless steel	117	-	Paclitaxel	Drug-eluting time: 40–50% in the first week, while the remainder is released by the end of 4 weeks	[31,114]
Achieve	Stainless steel		-	Paclitaxel	Drug-eluting time: 28% within 4 days; 69% within 2 weeks	[31]
Amazonia PAX	Cobalt-chromium L-605	73	-	Paclitaxel	Drug-eluting time: 60% within 2 days, while the remainder is released by the end of 7 weeks	[31,114]
Cre8	Cobalt-chromium L-605	70–80	-	Amphilimus	Drug-eluting time: 50% on the first day, while the remainder is released by the end of 3 weeks	[31,114]
BioFreedom	Stainless steel	119	-	Biolimus A9	Drug-eluting time: 98% within 4 weeks Outcomes at 1 year (percent from the total number of patients in the trial): - target vessel re-vascularization: 5.1% - stent thrombosis: 0% - cardiac mortality: 1.8%	[31,114,115]
JANUS	Stainless steel		Carbofilm	Tacrolimus	Drug-eluting time: 50% within the first 4 weeks Outcomes at 22 months (percent from the total number of patients in the trial): - target vessel re-vascularization: 32.3% - cardiac mortality: 5.5% - intraprocedural stent thrombosis: 2.1%	[31,116]
NANO +	Stainless steel	90	-	Sirolimus	Drug-eluting time: 85% during the first 4 weeks	[31,114]
BioMatri × Flex	Stainless steel	120	PLLA	Biolimus A9	Polymer coating degradation: 6 to 9 months	[113]
Endeavor	Cobalt-chromium MP35 N	91	Phosphorylcholine	Zotarolimus	Drug-eluting time: 80% during the first 10 days	[31,117,118]
Orsiro	Cobalt-chromium alloy	60	PLLA with silicon carbide layer	Sirolimus	Polymer coating degradation: 12 months Outcomes at 1 year (percent from the total number of patients in the trial): - target vessel re-vascularization: 3.6% - cardiac mortality: 0.8%	[113,119]
Synergy	Platinum-chromium	74	PLGA	Everolimus	Polymer coating degradation: 3 months	[113]
Promus Element	Platinum-chromium alloy	81	Permanent fluorinated polymer	Everolimus	Outcomes at 9 months (from total number of patients in the trial): - in-stent restenosis: 9% - stent fracture: 2.2%	[113,117,120]
MiStent	Cobalt-chromium alloy	64	PLGA	Sirolimus	Polymer coating degradation: 3 months	[113]
Mitsu	Cobalt-chromium alloy	40 × 80	Lipid nano-spheres	Merilimus	Polymer coating degradation: 1.5 months	[113]
Xience V	Cobalt-chromium L-605	81	Poly(vinyldenefluoride-co-hexafluoropropylene)	Everolimus	Drug-eluting time: 80% during first 30 days Outcomes at 1 year (percent from the total number of patients in the trial): - target vessel re-vascularization: 7.8% - stent thrombosis: 0.2% - cardiac mortality: 1.8%	[112,113,117]
Resolute Integrity	Cobalt-chromium alloy	91	BioLinx polymer	Zotarolimus	Outcomes at 1 year (percent from the total number of patients in the trial): - target vessel re-vascularization: 4.2% - cardiac mortality: 1.7%	[113,117,119]
Magmaris	Magnesium alloy	120–150	PLLA	Sirolimus	Resorption time: 12 months Drug-eluting time: 90 days	[83,121]
AMS 1.0	Magnesium alloy	165	-	-	Resorption time: <4 months	[113]
AMS 2.0	Magnesium alloy	120	-	-	Resorption time: >4 months	[113]
DREAMS 1	Magnesium alloy	125	PLGA	Paclitaxel	Resorption time: 9 months	[113]
DREAMS 2	Magnesium alloy	150	PLLA	Sirolimus	Resorption time: 9 months	[113]
Fantom	Tyrosine polycarbonate	125	-	Sirolimus	Resorption time: 36 months Outcomes at 1 year (percent from the total number of patients in the trial): - target lesion failure: 4.2% - stent thrombosis: 0.4%	[83,122]
ReZolve	Poly-tyrosine-derived polycarbonate	114–228	-	Sirolimus	Resorption time: 48 months	[113]
REVA Gen I	Poly-tyrosine-derived polycarbonate	200	-	Paclitaxel	Resorption time: 48 months	[113]
IDEAL BioStent Gen I	Polylactide anhydride mixed with a polymer of salycilic acid with a sebaic acid linker	200	Salicylate linked with adipic acid	Sirolimus	Resorption time: 6 to 9 months	[113]
Fortitude	PLLA	150	Sirolimus:polymer matrix (1:1)	Sirolimus	Drug-eluting time: 90% during first 90 days Resorption time: 10 months Higher mechanical strength, expansion capabilities, and resistance to fracture than other BVSs	[83,123]
MeRes 100	PLLA	100	PDLLA	Everolimus/Sirolimus	Resorption time: 24–36 months Drug-eluting time: 90 days	[83,124]
DESolve	PLLA	150	PLLA	Myolimus/Novolimus	Resorption time: 12 to 24 months	[83,113]
Magnitude	PLLA	<100	-	Sirolimus	Resorption time: 24–36 months	[83,125]
ABSORB BVS	PLLA	150	PDLLA	Everolimus	Resorption time: 36 months Drug-eluting time: 90 days Early and sustained safety in both simple lesions of stable patients and more complex anatomic and clinical settings	[83,124,126,127]

## Data Availability

Data sharing is not applicable to this article.

## References

[1] Kulkarni P., Rawtani D., Kumar M., Lahoti S.R. (2020). Cardiovascular drug delivery: A review on the recent advancements in nanocarrier based drug delivery with a brief emphasis on the novel use of magnetoliposomes and extracellular vesicles and ongoing clinical trial research. J. Drug Deliv. Sci. Technol..

[2] Bukala J., Buszman P.P., Małachowski J., Mazurkiewicz L., Sybilski K. (2020). Experimental Tests, FEM Constitutive Modeling and Validation of PLGA Bioresorbable Polymer for Stent Applications. Materials.

[3] Morciano G., Patergnani S., Bonora M., Pedriali G., Tarocco A., Bouhamida E., Marchi S., Ancora G., Anania G., Wieckowski M.R. (2020). Mitophagy in Cardiovascular Diseases. J. Clin. Med..

[4] (2019). Synthesis of new azaindeno-acetonitrile derivative with inotropic activity against heart failure model. Biointerface Res. Appl. Chem..

[5] (2020). Microscopic and submicroscopic structure of the heart atria and auricles in condition of the experimental thermal trauma. Biointerface Res. Appl. Chem..

[6] Ho M.-Y., Chen C.-C., Wang C.-Y., Chang S.-H., Hsieh M.-J., Lee C.-H., Wu V.C.-C., Hsieh I.-C. (2016). The Development of Coronary Artery Stents: From Bare-Metal to Bio-Resorbable Types. Metals.

[7] Canfield J., Totary-Jain H. (2018). 40 Years of Percutaneous Coronary Intervention: History and Future Directions. J. Pers. Med..

[8] Brown J.C., Gerhardt T.E., Kwon E. (2020). Risk Factors for Coronary Artery Disease.

[9] Emery C., Torreton E., Briere J.-B., Evers T., Fagnani F. (2020). Economic burden of coronary artery disease or peripheral artery disease in patients at high risk of ischemic events in the French setting: A claims database analysis. J. Med. Econ..

[10] Darba S., Safaei N., Mahboub–Ahari A., Nosratnejad S., Alizadeh G., Ameri H., Yousefi M. (2020). Direct and Indirect Costs Associated with Coronary Artery (Heart) Disease in Tabriz, Iran. Risk Manag. Healthc. Policy.

[11] Duhan N., Barak S., Mudgil D. (2020). Bioactive Lipids: Chemistry & Health Benefits. Biointerface Res. Appl. Chem..

[12] Khan W., Farah S., Domb A.J. (2012). Drug eluting stents: Developments and current status. J. Control. Release.

[13] Blake Y. (2020). Biocompatible materials for cardiovascular stents. Zenodo.

[14] Beshchasna N., Ho A.Y.K., Saqib M., Kraśkiewicz H., Wasyluk Ł., Kuzmin O., Duta O.C., Ficai D., Trusca R.D., Ficai A. (2019). Surface evaluation of titanium oxynitride coatings used for developing layered cardiovascular stents. Mater. Sci. Eng. C.

[15] Choubey R.K., Pradhan S.K. (2020). Prediction of strength and radial recoil of various stents using FE analysis. Mater. Today Proc..

[16] Chaparro-Rico B.D.M., Sebastiano F., Cafolla D. (2020). A Smart Stent for Monitoring Eventual Restenosis: Computational Fluid Dynamic and Finite Element Analysis in Descending Thoracic Aorta. Machines.

[17] Yelamanchili V.S., Hajouli S. (2020). Coronary Artery Stents.

[18] Cockerill I., See C.W., Young M.L., Wang Y., Zhu D. (2021). Designing Better Cardiovascular Stent Materials: A Learning Curve. Adv. Funct. Mater..

[19] Lee Y., Veerubhotla K., Jeong M.H., Lee C.H. (2019). Deep Learning in Personalization of Cardiovascular Stents. J. Cardiovasc. Pharmacol. Ther..

[20] Arafat M., Fouladian P., Blencowe A., Albrecht H., Song Y., Garg S. (2019). Drug-Eluting non-Vascular stents for localised drug targeting in obstructive gastrointestinal cancers. J. Control. Release.

[21] Ranade S.V., Miller K.M., Richard R.E., Chan A.K., Allen M.J., Helmus M.N. (2004). Physical characterization of controlled release of paclitaxel from the TAXUS? Express2? drug-Eluting stent. J. Biomed. Mater. Res..

[22] Schmidt T., Abbott J.D. (2018). Coronary Stents: History, Design, and Construction. J. Clin. Med..

[23] Ako J., Bonneau H.N., Honda Y., Fitzgerald P.J. (2007). Design Criteria for the Ideal Drug-Eluting Stent. Am. J. Cardiol..

[24] Fischell T.A., Balmuri A., Agarwal S., Verhye S. (2020). Integrated Stent Delivery System: A Next Generation of Stent Delivery and Drug-Eluting Stent. Cardiovasc. Revasc. Med..

[25] Pilgrim T., Muller O., Heg D., Roffi M., Kurz D.J., Moarof I., Weilenmann D., Kaiser C., Tapponnier M., Losdat S. (2021). Biodegradable-Versus Durable-Polymer Drug-Eluting Stents for STEMI. JACC Cardiovasc. Interv..

[26] Stefanini G.G., Holmes D.R. (2013). Drug-Eluting Coronary-Artery Stents. N. Engl. J. Med..

[27] Zhou C., Feng X., Shi Z., Song C., Cui X., Zhang J., Li T., Toft E.S., Ge J., Wang L. (2020). Research on elastic recoil and restoration of vessel pulsatility of Zn-Cu biodegradable coronary stents. Biomed. Tech. Eng..

[28] Iqbal J., Gunn J., Serruys P.W. (2013). Coronary stents: Historical development, current status and future directions. Br. Med. Bull..

[29] Montero-Baker M., Braun J.D., Weinkauf C., Leon L.R., Latifi R., Rhee P., Gruessner R.W.G. (2015). Technological Advances in Endovascular Surgery. Technological Advances in Surgery, Trauma and Critical Care.

[30] Hermawan H., Mantovani D. (2013). Process of prototyping coronary stents from biodegradable Fe–Mn alloys. Acta Biomater..

[31] Beshchasna N., Saqib M., Kraskiewicz H., Wasyluk Ł., Kuzmin O., Duta O.C., Ficai D., Ghizdavet Z., Marin A., Ficai A. (2020). Recent Advances in Manufacturing Innovative Stents. Pharmaceutics.

[32] Yue R., Niu J., Li Y., Ke G., Huang H., Pei J., Ding W., Yuan G. (2020). In Vitro cytocompatibility, hemocompatibility and antibacterial properties of biodegradable Zn-Cu-Fe alloys for cardiovascular stents applications. Mater. Sci. Eng. C.

[33] Saraf A.R., Yadav S.P., Wall J.G., Podbielska H., Wawrzyńska M. (2018). 2-Fundamentals of bare-metal stents. Functionalised Cardiovascular Stents.

[34] Azaouzi M., Lebaal N., Makradi A., Belouettar S. (2013). Optimization based simulation of self-expanding Nitinol stent. Mater. Des..

[35] Mwangi J.W., Nguyen L.T., Bui V.D., Berger T., Zeidler H., Schubert A. (2019). Nitinol manufacturing and micromachining: A review of processes and their suitability in processing medical-grade nitinol. J. Manuf. Process..

[36] Schuessler A., Bayer U., Siekmeyer G., Steegmueller R., Strobel M., Schuessler A. Manufacturing of stents: Optimize the stent with new manufacturing technologies. Proceedings of the 5th European Symposium of Vascular Biomaterials ESVB.

[37] Obayi C.S., Tolouei R., Mostavan A., Paternoster C., Turgeon S., Okorie B.A., Obikwelu D.O., Mantovani D. (2016). Effect of grain sizes on mechanical properties and biodegradation behavior of pure iron for cardiovascular stent application. Biomatter.

[38] Grogan J., Leen S., McHugh P. (2012). Comparing coronary stent material performance on a common geometric platform through simulated bench testing. J. Mech. Behav. Biomed. Mater..

[39] Grogan J.A., Leen S.B., McHugh P.E. (2013). Optimizing the design of a bioabsorbable metal stent using computer simulation methods. Biomaterials.

[40] Alicea L.A., Aviles J.I., López I.A., Mulero L.E., Sánchez L.A. Mechanics biomaterials: Stents, 2004. Course Materials in the Department of General Engineering, University of Puerto Rico, Mayaguez. https://blogs.epfl.ch/stents/documents/biomechanics%20of%20stents.pdf.

[41] Brailovski V., Prokoshkin S., Gauthier M., Inaekyan K., Dubinskiy S., Petrzhik M., Filonov M. (2011). Bulk and porous metastable beta Ti–Nb–Zr(Ta) alloys for biomedical applications. Mater. Sci. Eng. C.

[42] Poncin P., Millet C., Chevy J., Proft J.L. Comparing and optimizing Co-Cr tubing for stent applications. Proceedings of the Material and Processes for Medical Devices Conference.

[43] Commandeur S., Van Beusekom H.M., Van Der Giessen W.J. (2006). Polymers, drug release, and drug-eluting stents. J. Interv. Cardiol..

[44] Qian M., Liu Q., Wei Y., Guo Z., Zhao Q. (2021). In-Situ biotransformation of nitric oxide by functionalized surfaces of cardiovascular stents. Bioact. Mater..

[45] Tsai M.-L., Hsieh M.-J., Chen C.-C., Chang S.-H., Wang C.-Y., Chen D.-Y., Yang C.-H., Yeh J.-K., Ho M.-Y., Hsieh I.-C. (2020). Comparison of 9-Month Angiographic Follow-Up and Long-Term Clinical Outcomes of Biodegradable Polymer Drug-Eluting Stents and Second-Generation Durable Polymer Drug-Eluting Stents in Patients Undergoing Single Coronary Artery Stenting. Acta Cardiol. Sin..

[46] Pendyala L.K., Yin X., Li J., Chen J.P., Chronos N., Hou D. (2009). The First-Generation Drug-Eluting Stents and Coronary Endothelial Dysfunction. JACC Cardiovasc. Interv..

[47] Ali R.M., Kader M.A.S.A., Ahmad W.A.W., Ong T.K., Liew H.B., Omar A.-F., Zuhdi A.S.M., Nuruddin A.A., Schnorr B., Scheller B. (2019). Treatment of Coronary Drug-Eluting Stent Restenosis by a Sirolimus- or Paclitaxel-Coated Balloon. JACC Cardiovasc. Interv..

[48] Chen Y., Zeng Y., Zhu X., Miao L., Liang X., Duan J., Li H., Tian X., Pang L., Wei Y. (2021). Significant difference between sirolimus and paclitaxel nanoparticles in anti-proliferation effect in normoxia and hypoxia: The basis of better selection of atherosclerosis treatment. Bioact. Mater..

[49] Kobo O., Saada M., Meisel S.R., Hellou E., Frimerman A., Abu Fanne R., Mohsen J., Danon A., Roguin A. (2020). Modern Stents: Where Are We Going?. Rambam Maimonides Med. J..

[50] Kalachev L.V. (2016). Modelling Simple Experimental Platform for In Vitro Study of Drug Elution from Drug Eluting Stents (DES). J. Phys. Conf. Ser..

[51] Byrne R.A., Joner M., Kastrati A. (2015). Stent thrombosis and restenosis: What have we learned and where are we going? The Andreas Grüntzig Lecture ESC 2014. Eur. Heart J..

[52] Seidlitz A., Fotaki N., Klein S. (2019). Drug-Eluting Stents. In Vitro Drug Release Testing of Special Dosage Forms.

[53] Martin D.M., Boyle F.J. (2011). Drug-Eluting stents for coronary artery disease: A review. Med Eng. Phys..

[54] Vale N., Madeira S., Almeida M., Raposo L., Freitas P., Castro M., Rodrigues G., Oliveira A., Brito J., Leal S. (2020). Ten-year survival of patients undergoing coronary angioplasty with first-generation sirolimus-eluting stents and bare-metal stents. Rev. Port. Cardiol..

[55] Sambola A., Rello P., Soriano T., Bhatt D.L., Pasupuleti V., Cannon C.P., Gibson C.M., Dewilde W.J., Lip G.Y., Peterson E.D. (2020). Safety and efficacy of drug eluting stents vs bare metal stents in patients with atrial fibrillation: A systematic review and meta-analysis. Thromb. Res..

[56] Choi J.M., Lee S.-H., Kang M., Choi J.-H. (2020). Impact of medication adherence to dual antiplatelet therapy on the long-term outcome of drug-Eluting or bare-Metal stents. PLoS ONE.

[57] Montalescot G., Brieger D., Dalby A.J., Park S.-J., Mehran R. (2015). Duration of Dual Antiplatelet Therapy After Coronary Stenting. J. Am. Coll. Cardiol..

[58] Levine G.N., Bates E.R., Blankenship J.C., Bailey S.R., Bittl J.A., Cercek B., Chambers C.E., Ellis S.G., Guyton R.A., Hollenberg S.M. (2011). 2011 ACCF/AHA/SCAI Guideline for Percutaneous Coronary Intervention. Circulation.

[59] Members T.F., Windecker S., Kolh P., Alfonso F., Collet J.-P., Cremer J., Falk V., Filippatos G., Hamm C.W., Head S.J. (2014). 2014 ESC/EACTS Guidelines on myocardial revascularization. Eur. Heart J..

[60] Kereiakes D.J., Yeh R.W., Massaro J.M., Driscoll-Shempp P., Cutlip D.E., Steg P.G., Gershlick A.H., Darius H., Meredith I.T., Ormiston J. (2015). Antiplatelet Therapy Duration Following Bare Metal or Drug-Eluting Coronary Stents. JAMA.

[61] Otsuka F., Nakano M., Ladich E., Kolodgie F.D., Virmani R. (2012). Pathologic Etiologies of Late and Very Late Stent Thrombosis following First-Generation Drug-Eluting Stent Placement. Thrombosis.

[62] De Luca G., Smits P., Hofma S.H., Di Lorenzo E., Vlachojannis G.J., Hof A.W.V., van Boven A.J., Kedhi E., Stone G.W., Suryapranata H. (2017). Everolimus eluting stent vs. first generation drug-Eluting stent in primary angioplasty: A pooled patient-Level meta-Analysis of randomized trials. Int. J. Cardiol..

[63] Partida R.A., Yeh R.W. (2017). Contemporary drug-Eluting stent platforms: Design, safety, and clinical efficacy. Cardiol. Clin..

[64] Liu J., Wang J., Xue Y.-F., Chen T.-T., Huang D.-N., Wang Y.-X., Ren K.-F., Wang Y.-B., Fu G.-S., Ji J. (2020). Biodegradable phosphorylcholine copolymer for cardiovascular stent coating. J. Mater. Chem. B.

[65] Nakayama Y., Nishi S., Ishibashi-Ueda H. (2003). Fabrication of drug-Eluting covered stents with micropores and differential coating of heparin and FK506. Cardiovasc. Radiat. Med..

[66] Livingston M., Tan A. (2015). Coating Techniques and Release Kinetics of Drug-Eluting Stents. J. Med. Devices.

[67] Grabow N., Schmitt L., Pfensig S., Reske T., Rehme H., Senz V., Sternberg K., Schmitz K.-P. (2012). Spray-Coating process development, manufacture, quality assessment and drug release behavior of peripheral drug-eluting stents. Biomed. Tech. Eng..

[68] Bundhun P.K., Yanamala C.M., Huang W.-Q. (2017). Comparing Stent Thrombosis associated with Zotarolimus Eluting Stents versus Everolimus Eluting Stents at 1 year follow up: A systematic review and meta-analysis of 6 randomized controlled trials. BMC Cardiovasc. Disord..

[69] Roleder T., Kedhi E., Berta B., Gasior P., Wanha W., Roleder M., Fluder J., Smolka G., Ochala A., Wojakowski W. (2019). Short-term stent coverage of second-Generation zotarolimus-Eluting durable polymer stents: Onyx one-Month optical coherence tomography study. Adv. Interv. Cardiol..

[70] Kalra A., Rehman H., Khera S., Thyagarajan B., Bhatt D.L., Kleiman N.S., Yeh R.W. (2017). New-Generation Coronary Stents: Current Data and Future Directions. Curr. Atheroscler. Rep..

[71] Kawashima H., Zocca P., Buiten R.A., Smits P.C., Onuma Y., Wykrzykowska J.J., De Winter R.J., Von Birgelen C., Serruys P.W. (2020). The 2010s in clinical drug-Eluting stent and bioresorbable scaffold research: A Dutch perspective. Neth. Heart J..

[72] Parker W., Iqbal J., Trust S.S.T.H.N.F. (2020). Comparison of Contemporary Drug-Eluting Coronary Stents–Is Any Stent Better than the Others?. Heart Int..

[73] Hwang C.-W., Wu D., Edelman E.R. (2003). Impact of transport and drug properties on the local pharmacology of drug-Eluting stents. Int. J. Cardiovasc. Interv..

[74] Escuer J., Cebollero M., Peña E., McGinty S., Martínez M.A. (2020). How does stent expansion alter drug transport properties of the arterial wall?. J. Mech. Behav. Biomed. Mater..

[75] Sakamoto A., Jinnouchi H., Torii S., Virmani R., Finn A.V. (2018). Understanding the Impact of Stent and Scaffold Material and Strut Design on Coronary Artery Thrombosis from the Basic and Clinical Points of View. Bioengineering.

[76] Tan J., Cui Y., Zeng Z., Wei L., Li L., Wang H., Hu H., Liu T., Huang N., Chen J. (2020). Heparin/poly-l-lysine nanoplatform with growth factor delivery for surface modification of cardiovascular stents: The influence of vascular endothelial growth factor loading. J. Biomed. Mater. Res. Part A.

[77] Nappi F., Nenna A., Larobina D., Martuscelli G., Singh S.S.A., Chello M., Ambrosio L. (2021). The Use of Bioactive Polymers for Intervention and Tissue Engineering: The New Frontier for Cardiovascular Therapy. Polymers.

[78] Aljihmani L., Alic L., Boudjemline Y., Hijazi Z.M., Mansoor B., Serpedin E., Qaraqe K. (2019). Magnesium-Based Bioresorbable Stent Materials: Review of Reviews. J. Bio Tribo Corros..

[79] Wang C., Zhang L., Fang Y., Sun W. (2020). Design, Characterization, and 3D Printing of Cardiovascular Stents with Zero Poisson’s Ratio in Longitudinal Deformation. Engineering.

[80] Schieber R., Raymond Y., Caparrós C., Bou J., Acero E.H., Guebitz G., Canal C., Pegueroles M. (2021). Functionalization Strategies and Fabrication of Solvent-Cast PLLA for Bioresorbable Stents. Appl. Sci..

[81] Waksman R. (2006). Biodegradable stents: They do their job and disappear. J. Invasive Cardiol..

[82] Toong D.W.Y., Ng J.C.K., Huang Y., Wong P.E.H., Leo H.L., Venkatraman S.S., Ang H.Y. (2020). Bioresorbable metals in cardiovascular stents: Material insights and progress. Materialia.

[83] Omar W.A., Kumbhani D.J. (2019). The Current Literature on Bioabsorbable Stents: A Review. Curr. Atheroscler. Rep..

[84] Fu J., Su Y., Qin Y.-X., Zheng Y., Wang Y., Zhu D. (2020). Evolution of metallic cardiovascular stent materials: A comparative study among stainless steel, magnesium and zinc. Biomaterials.

[85] Koo Y., Tiasha T., Shanov V.N., Yun Y. (2017). Expandable Mg-Based Helical Stent Assessment using Static, Dynamic, and Porcine Ex Vivo Models. Sci. Rep..

[86] Wang J., Giridharan V., Shanov V., Xu Z., Collins B., White L., Jang Y., Sankar J., Huang N., Yun Y. (2014). Flow-Induced corrosion behavior of absorbable magnesium-Based stents. Acta Biomater..

[87] Paryab N., Cronin D., Lee-Sullivan P., Ying X., Boey F.Y.C., Venkatraman S., Venkatraman S. (2012). Uniform Expansion of a Polymeric Helical Stent. J. Med. Devices.

[88] Su S.-H., Chao R.Y.N., Landau C.L., Nelson K.D., Timmons R.B., Meidell R.S., Eberhart R.C. (2003). Expandable bioresorbable endovascular stent. I. Fabrication and properties. Ann. Biomed. Eng..

[89] Zhao F., Sun J., Xue W., Wang F., King M.W., Yu C., Jiao Y., Sun K., Wang L. (2021). Development of a polycaprolactone/poly(p-dioxanone) bioresorbable stent with mechanically self-reinforced structure for congenital heart disease treatment. Bioact. Mater..

[90] Sharma U., Concagh D., Core L., Kuang Y., You C., Pham Q., Zugates G., Busold R., Webber S., Merlo J. (2017). The development of bioresorbable composite polymeric implants with high mechanical strength. Nat. Mater..

[91] Zhao F., Xue W., Wang F., Sun J., Lin J., Liu L., Sun K., Wang L. (2019). Braided bioresorbable cardiovascular stents mechanically reinforced by axial runners. J. Mech. Behav. Biomed. Mater..

[92] Han X., Wu X., Kelly M., Chen X. (2017). Fabrication and Optimal Design of Biodegradable Polymeric Stents for Aneurysms Treatments. J. Funct. Biomater..

[93] Park S.A., Lee S.J., Lim K.S., Bae I.H., Lee J.H., Kim W.D., Jeong M.H., Park J.-K. (2015). In Vivo evaluation and characterization of a bio-Absorbable drug-Coated stent fabricated using a 3D-Printing system. Mater. Lett..

[94] Van Lith R., Baker E., Ware H., Yang J., Farsheed A.C., Sun C., Ameer G. (2016). 3D-Printing Strong High-Resolution Antioxidant Bioresorbable Vascular Stents. Adv. Mater. Technol..

[95] Liu Y., Xiang K., Li Y., Chen H., Hu Q. (2014). Combining 3D Printing and Electrospinning for the Fabrication of a Bioabsorbable Poly-p-dioxanone Stent. Adv. Transdiscipl. Eng..

[96] Lee C.-H., Hsieh M.-J., Liu S.-C., Chen J.-K., Liu S.-J., Hsieh I.-C., Wen M.-S., Hung K.-C. (2018). Novel bifurcation stents coated with bioabsorbable nanofibers with extended and controlled release of rosuvastatin and paclitaxel. Mater. Sci. Eng. C.

[97] Mostaed E., Sikora-Jasinska M., Loffredo S., Demir A., Previtali B., Mantovani D., Beanland R., Vedani M. (2016). Novel Zn-Based alloys for biodegradable stent applications: Design, development and in vitro degradation. J. Mech. Behav. Biomed. Mater..

[98] Flege C., Vogt F., Höges S., Jauer L., Borinski M., Schulte V.A., Hoffmann R., Poprawe R., Meiners W., Jobmann M. (2013). Development and characterization of a coronary polylactic acid stent prototype generated by selective laser melting. J. Mater. Sci. Mater. Electron..

[99] Ang H.Y., Huang Y.Y., Lim S.T., Wong P., Joner M., Foin N. (2017). Mechanical behavior of polymer-based vs. metallic-based bioresorbable stents. J. Thorac. Dis..

[100] Montava-Jorda S., Chacon V., Lascano D., Sanchez-Nacher L., Montanes N. (2019). Manufacturing and Characterization of Functionalized Aliphatic Polyester from Poly(lactic acid) with Halloysite Nanotubes. Polymers.

[101] Wang Q., Fang G., Zhao Y.-H., Zhou J. (2018). Improvement of Mechanical Performance of Bioresorbable Magnesium Alloy Coronary Artery Stents through Stent Pattern Redesign. Appl. Sci..

[102] Chang F.-Y., Chen Y.-C., Liang T.-H., Cai Z.-Y. (2020). Fabrication of Edge Rounded Polylactic Acid Biomedical Stents by the Multi-Axis Micro-Milling Process. Appl. Sci..

[103] Borhani S., Hassanajili S., Tafti S.H.A., Rabbani S. (2018). Cardiovascular stents: Overview, evolution, and next generation. Prog. Biomater..

[104] Onuma Y., Serruys P. (2011). Bioresorbable Scaffold. Circulation.

[105] Tesfamariam B. (2016). Bioresorbable vascular scaffolds: Biodegradation, drug delivery and vascular remodeling. Pharmacol. Res..

[106] Das D., Zhang Z., Winkler T., Mour M., Günter C.I., Morlock M.M., Machens H.-G., Schilling A.F. (2011). Bioresorption and Degradation of Biomaterials. Adv. Biochem. Eng. Biotechnol..

[107] Moravej M., Mantovani D. (2011). Biodegradable Metals for Cardiovascular Stent Application: Interests and New Opportunities. Int. J. Mol. Sci..

[108] Chen C., Chen J., Wu W., Shi Y., Jin L., Petrini L., Shen L., Yuan G., Ding W., Ge J. (2019). In Vivo and in Vitro evaluation of a biodegradable magnesium vascular stent designed by shape optimization strategy. Biomaterials.

[109] Kang M.-H., Cheon K.-H., Jo K.-I., Ahn J.-H., Kim H.-E., Jung H.-D., Jang T.-S. (2020). An asymmetric surface coating strategy for improved corrosion resistance and vascular compatibility of magnesium alloy stents. Mater. Des..

[110] Kandala B.S.P.K., Zhang G., Hopkins T.M., An X., Pixley S.K., Shanov V. (2019). In Vitro and In Vivo Testing of Zinc as a Biodegradable Material for Stents Fabricated by Photo-Chemical Etching. Appl. Sci..

[111] Maeng M., Jensen L.O., Falk E., Andersen H.R., Thuesen L. (2008). Negative vascular remodelling after implantation of bioabsorbable magnesium alloy stents in porcine coronary arteries: A randomised comparison with bare-metal and sirolimus-eluting stents. Heart.

[112] Waksman R., Barbash I.M., Dvir D., Torguson R., Ben-Dor I., Maluenda G., Xue Z., Satler L.F., Suddath W.O., Kent K.M. (2012). Safety and Efficacy of the XIENCE V Everolimus-Eluting Stent Compared to First-Generation Drug-Eluting Stents in Contemporary Clinical Practice. Am. J. Cardiol..

[113] Foin N., Lee R.D., Torii R., Guitierrez-Chico J.L., Mattesini A., Nijjer S., Sen S., Petraco R., Davies J.E., Di Mario C. (2014). Impact of stent strut design in metallic stents and biodegradable scaffolds. Int. J. Cardiol..

[114] Baquet M., Jochheim D., Mehilli J. (2018). Polymer-Free drug-Eluting stents for coronary artery disease. J. Interv. Cardiol..

[115] Costa R.A., Abizaid A., Mehran R., Schofer J., Schuler G.C., Hauptmann K.E., Magalhães M.A., Parise H., Grube E. (2016). Polymer-Free Biolimus A9-Coated Stents in the Treatment of De Novo Coronary Lesions. JACC Cardiovasc. Interv..

[116] Tamburino C., Di Salvo M.E., Capodanno D., Capranzano P., Parisi R., Mirabella F., Scardaci F., Ussia G., Galassi A.R., Fiscella D. (2008). Real world safety and efficacy of the Janus tacrolimus-eluting stent: Long-term clinical outcome and angiographic findings from the tacrolimus-eluting stent (TEST) registry. Catheter. Cardiovasc. Interv..

[117] El-Hayek G., Bangalore S., Dominguez A.C., Devireddy C., Jaber W., Kumar G., Mavromatis K., Tamis-Holland J., Samady H. (2017). Meta-Analysis of Randomized Clinical Trials Comparing Biodegradable Polymer Drug-Eluting Stent to Second-Generation Durable Polymer Drug-Eluting Stents. JACC Cardiovasc. Interv..

[118] Medtronic (2007). Endeavor (R) Drug-Eluting Coronary Stent.

[119] Kim S., Kang S., Lee J.M., Chung W., Park J.J., Yoon C., Suh J., Cho Y., Doh J., Cho J.M. (2020). Three-Year clinical outcome of biodegradable hybrid polymer Orsiro sirolimus-eluting stent and the durable biocompatible polymer Resolute Integrity zotarolimus-eluting stent: A randomized controlled trial. Catheter. Cardiovasc. Interv..

[120] Kuramitsu S., Hiromasa T., Enomoto S., Shinozaki T., Iwabuchi M., Mazaki T., Domei T., Yamaji K., Soga Y., Hyodo M. (2015). Incidence and Clinical Impact of Stent Fracture After PROMUS Element Platinum Chromium Everolimus-Eluting Stent Implantation. JACC Cardiovasc. Interv..

[121] Rapetto C., Leoncini M. (2017). Magmaris: A new generation metallic sirolimus-Eluting fully bioresorbable scaffold: Present status and future perspectives. J. Thorac. Dis..

[122] Chevalier B., Abizaid A., Carrié D., Frey N., Lutz M., Weber-Albers J., Dudek D., Weng S.-C., Akodad M., Anderson J. (2019). Clinical and Angiographic Outcomes With a Novel Radiopaque Sirolimus-Eluting Bioresorbable Vascular Scaffold. Circ. Cardiovasc. Interv..

[123] Chieffo A., Khawaja S.A., Vesga B., Hernandez H., Moncada M., Delgado J.A., Esposito G., Ferrone M., Dager A., Arana C. (2020). First in human evaluation of a novel Sirolimus-eluting ultra-high molecular weight bioresorbable scaffold: 9-, 24-and 36-months imaging and clinical results from the multi-center renascent study. Int. J. Cardiol..

[124] Lee D.-H., Hernandez J.M.D.L.T. (2018). The Newest Generation of Drug-eluting Stents and Beyond. Eur. Cardiol. Rev..

[125] Regazzoli D., Leone P.P., Colombo A., Latib A. (2017). New generation bioresorbable scaffold technologies: An update on novel devices and clinical results. J. Thorac. Dis..

[126] Kereiakes D.J., Ellis S.G., Metzger C., Caputo R.P., Rizik D.G., Teirstein P.S., Litt M.R., Kini A., Kabour A., Marx S.O. (2017). 3-Year Clinical Outcomes With Everolimus-Eluting Bioresorbable Coronary Scaffolds. J. Am. Coll. Cardiol..

[127] Rizik D.G., Hermiller J.B., Kereiakes D.J. (2015). The ABSORB bioresorbable vascular scaffold: A novel, fully resorbable drug-eluting stent: Current concepts and overview of clinical evidence. Catheter. Cardiovasc. Interv..

[128] Guerra A.J., San J., Ciurana J. (2017). Fabrication of PCL/PLA Composite Tube for Stent Manufacturing. Procedia CIRP.

[129] Chichareon P., Katagiri Y., Asano T., Takahashi K., Kogame N., Modolo R., Tenekecioglu E., Chang C.-C., Tomaniak M., Kukreja N. (2019). Mechanical properties and performances of contemporary drug-eluting stent: Focus on the metallic backbone. Expert Rev. Med Devices.

[130] Bink N., Mohan V.B., Fakirov S. (2021). Recent advances in plastic stents: A comprehensive review. Int. J. Polym. Mater..

[131] Wholey M.H., Finol E.A. (2007). Designing the ideal stent. Endovasc. Today.

[132] Watson T.I., Webster M.W., Ormiston J., Ruygrok P.N., Stewart J.T. (2017). Long and short of optimal stent design. Open Heart.

[133] Doenst T., Haverich A., Serruys P., Bonow R.O., Kappetein P., Falk V., Velazquez E., Diegeler A., Sigusch H. (2019). PCI and CABG for Treating Stable Coronary Artery Disease. J. Am. Coll. Cardiol..

[134] Dola J., Morawiec B., Muzyk P., Nowalany-Kozielska E., Kawecki D. (2020). Ideal coronary stent: Development, characteristics, and vessel size impact. Ann. Acad. Med. Silesiensis.

[135] Bowen P.K., Shearier E.R., Zhao S., Ii R.J.G., Zhao F., Goldman J., Drelich J.W. (2016). Biodegradable Metals for Cardiovascular Stents: From Clinical Concerns to Recent Zn-Alloys. Adv. Healthc. Mater..

[136] Nazneen F., Herzog G., Arrigan D.W., Caplice N., Benvenuto P., Galvin P., Thompson M. (2012). Surface chemical and physical modification in stent technology for the treatment of coronary artery disease. J. Biomed. Mater. Res. Part B Appl. Biomater..

[137] Polanec B., Kramberger J., Glodez S. (2020). A review of production technologies and materials for manufacturing of cardiovascular stents. Adv. Prod. Eng. Manag..

[138] Rab T., Abbott J.D., Basir M.B., Latib A., Kumar G., Meraj P., Croce K., Davé R. (2020). Summary of Practice Considerations for Percutaneous Coronary Intervention of Left Main Bifurcation Disease. Heart Int..

[139] Gil R.J., Kern A., Pawłowski T., Bil J. (2020). Twelve-Month clinical results from the new cobalt-chromium sirolimus-eluting dedicated bifurcation stent BiOSS LIM C Registry. Arch. Med. Sci..

[140] Mishra S. (2016). Dedicated bifurcation stents–Mechanistic, hardware, and technical aspects. Indian Heart J..

[141] Tan S., Ramzy J., Burgess S., Zaman S. (2020). Percutaneous Coronary Intervention for Coronary Bifurcation Lesions: Latest Evidence. Curr. Treat. Options Cardiovasc. Med..

[142] Bowen P.K., Guillory R.J., Shearier E.R., Seitz J.-M., Drelich J., Bocks M., Zhao F., Goldman J. (2015). Metallic zinc exhibits optimal biocompatibility for bioabsorbable endovascular stents. Mater. Sci. Eng. C.

[143] Yang H., Wang C., Liu C., Chen H., Wu Y., Han J., Jia Z., Lin W., Zhang D., Li W. (2017). Evolution of the degradation mechanism of pure zinc stent in the one-year study of rabbit abdominal aorta model. Biomaterials.

[144] Huang L., Pu C., Fisher R.K., Mountain D.J., Gao Y., Liaw P.K., Zhang W., He W. (2015). A Zr-Based bulk metallic glass for future stent applications: Materials properties, finite element modeling, and In Vitro human vascular cell response. Acta Biomater..

[145] Miskovic D.M., Laws K.J., Ferry M., Wen C. (2021). 9-Metallic glasses. Structural Biomaterials.

[146] Hasannaeimi V., Sadeghilaridjani M., Mukherjee S. (2021). Electrochemical and Corrosion Behavior of Metallic Glasses.

[147] Kiani F., Wen C., Li Y. (2020). Prospects and strategies for magnesium alloys as biodegradable implants from crystalline to bulk metallic glasses and composites—A review. Acta Biomater..

[148] Motru S., Sachidananda M., Avyaktha K., Kumar G.P., Patil N. (2020). Structural analysis of bulk metallic glass cardio-vascular stent under dynamic and failure criteria. Mater. Today Proc..

[149] Kumar G.P., Tavakoli R., Cui F., Jafary-Zadeh M. (2016). Feasibility of using bulk metallic glass for self-expandable stent applications. J. Biomed. Mater. Res. Part B: Appl. Biomater..

[150] Jafary-Zadeh M., Kumar G.P., Branicio P.S., Seifi M., Lewandowski J.J., Cui F. (2018). A Critical Review on Metallic Glasses as Structural Materials for Cardiovascular Stent Applications. J. Funct. Biomater..

[151] Xie G., Wang X., Setsuhara Y., Kamiya T., Yamaura S.-I. (2019). Metallic Glasses for Biomedical Applications. Novel Structured Metallic and Inorganic Materials.

[152] Meagher P., O’Cearbhaill E.D., Byrne J.H., Browne D.J. (2016). Bulk Metallic Glasses for Implantable Medical Devices and Surgical Tools. Adv. Mater..

[153] Liu Y., Wang H.-J., Pang S.-J., Zhang T. (2020). Ti–Zr–Cu–Fe–Sn–Si–Ag–Ta bulk metallic glasses with good corrosion resistance as potential biomaterials. Rare Met..

[154] Yeazel T.R., Becker M.L. (2020). Advancing Toward 3D Printing of Bioresorbable Shape Memory Polymer Stents. Biomacromolecules.

[155] Kapoor D. (2017). Nitinol for Medical Applications: A Brief Introduction to the Properties and Processing of Nickel Titanium Shape Memory Alloys and their Use in Stents. Johns. Matthey Technol. Rev..

[156] Jia H., Gu S.-Y., Chang K. (2018). 3D printed self-expandable vascular stents from biodegradable shape memory polymer. Adv. Polym. Technol..

[157] Omid S.O., Goudarzi Z., Kangarshahi L.M., Mokhtarzade A., Bahrami F. (2020). Self-expanding stents based on shape memory alloys and shape memory polymers. J. Compos. Compd..

[158] Holman H., Kavarana M.N., Rajab T.K. (2021). Smart materials in cardiovascular implants: Shape memory alloys and shape memory polymers. Artif. Organs.

[159] Kato H., Fukushima S., Sasaki K., Sun Q., Matsui R., Takeda K., Pieczyska E.A. (2017). Shape Memory Effect and Superelasticity of Textured NiTi Alloy Wire. Advances in Shape Memory Materials: In Commemoration of the Retirement of Professor Hisaaki Tobushi.

[160] Ghobadi E., Shutov A., Steeb H. (2021). Parameter Identification and Validation of Shape-Memory Polymers within the Framework of Finite Strain Viscoelasticity. Materials.

[161] Zare M., Davoodi P., Ramakrishna S. (2021). Electrospun Shape Memory Polymer Micro-/Nanofibers and Tailoring Their Roles for Biomedical Applications. Nanomaterials.

[162] Shie M.-Y., Shen Y.-F., Astuti S.D., Lee A.K.-X., Lin S.-H., Dwijaksara N.L.B., Chen Y.-W. (2019). Review of Polymeric Materials in 4D Printing Biomedical Applications. Polymers.

[163] Basit A., L’Hostis G., Durand B. (2019). The recovery properties under load of a shape memory polymer composite material. Mater. Werkst..

[164] Mather P.T., Luo X., Rousseau I.A. (2009). Shape Memory Polymer Research. Annu. Rev. Mater. Res..

[165] Lee A.Y., An J., Chua C.K. (2017). Two-Way 4D Printing: A Review on the Reversibility of 3D-Printed Shape Memory Materials. Engineering.

[166] Chu C., Xiang Z., Wang J., Xie H., Xiang T., Zhou S. (2020). A near-infrared light-triggered shape-memory polymer for long-time fluorescence imaging in deep tissues. J. Mater. Chem. B.

[167] Huang W.M., Zhao Y., Wang C.C., Ding Z., Purnawali H., Tang C., Zhang J.L. (2012). Thermo/chemo-responsive shape memory effect in polymers: A sketch of working mechanisms, fundamentals and optimization. J. Polym. Res..

[168] Svedman C., Bruze M., Johansen J., Mahler V., Lepoittevin J.P., Frosch P. (2019). Coronary Stents. Contact Dermatitis.

[169] Nezami F.R., Athanasiou L.S., Edelman E.R., Ohayon J., Finet G., Pettigrew R.I. (2021). Chapter 28-Endovascular drug-delivery and drug-elution systems. Biomechanics of Coronary Atherosclerotic Plaque.

[170] Worthley S.G., Abizaid A., Kirtane A.J., Simon D.I., Windecker S., Brar S., Meredith I.T., Shetty S., Sinhal A., Almonacid A.P. (2017). First-in-Human Evaluation of a Novel Polymer-Free Drug-Filled Stent. JACC Cardiovasc. Interv..

[171] Yerasi C., Case B.C., Forrestal B.J., Torguson R., Weintraub W.S., Garcia-Garcia H.M., Waksman R. (2020). Drug-Coated Balloon for De Novo Coronary Artery Disease. J. Am. Coll. Cardiol..

[172] Giacoppo D., Alfonso F., Xu B., Claessen B.E., Adriaenssens T., Jensen C., Pérez-Vizcayno M.J., Kang D.-Y., Degenhardt R., Pleva L. (2020). Drug-Coated Balloon Angioplasty Versus Drug-Eluting Stent Implantation in Patients with Coronary Stent Restenosis. J. Am. Coll. Cardiol..

[173] Fahrni G., Scheller B., Coslovsky M., Gilgen N., Farah A., Ohlow M.-A., Mangner N., Weilenmann D., Wöhrle J., Cuculi F. (2020). Drug-coated balloon versus drug-eluting stent in small coronary artery lesions: Angiographic analysis from the BASKET-SMALL 2 trial. Clin. Res. Cardiol..

[174] Majewska P., Oledzka E., Sobczak M. (2019). Overview of the latest developments in the field of drug-eluting stent technology. Biomater. Sci..

[175] Schieber R., Lasserre F., Hans M., Fernández-Yagüe M., Díaz-Ricart M., Escolar G., Ginebra M.-P., Mücklich F., Pegueroles M. (2017). Direct Laser Interference Patterning of CoCr Alloy Surfaces to Control Endothelial Cell and Platelet Response for Cardiovascular Applications. Adv. Healthc. Mater..

[176] Lee S.J., Jo H.H., Lim K.S., Lim D., Lee S., Lee J.H., Kim W.D., Jeong M.H., Lim J.Y., Kwon I.K. (2019). Heparin coating on 3D printed poly (l-lactic acid) biodegradable cardiovascular stent via mild surface modification approach for coronary artery implantation. Chem. Eng. J..

[177] Diaz-Rodriguez S., Rasser C., Mesnier J., Chevallier P., Gallet R., Choqueux C., Even G., Sayah N., Chaubet F., Nicoletti A. (2021). Coronary stent CD31-mimetic coating favours endothelialization and reduces local inflammation and neointimal development in vivo. Eur. Heart J..

[178] He X., Zhang G., Pei Y., Zhang H. (2020). Layered hydroxide/polydopamine/hyaluronic acid functionalized magnesium alloys for enhanced anticorrosion, biocompatibility and antithrombogenicity in vascular stents. J. Biomater. Appl..

[179] Park K.-S., Kang S.N., Kim D.H., Kim H.-B., Im K.S., Park W., Hong Y.J., Han D.K., Joung Y.K. (2020). Late endothelial progenitor cell-capture stents with CD146 antibody and nanostructure reduce in-stent restenosis and thrombosis. Acta Biomater..

[180] Blanco E., Segura-Ibarra V., Bawa D., Wu S., Liu H., Ferrari M., Lumsden A.B., Shah D.J., Lin C.H. (2019). Functionalization of endovascular devices with superparamagnetic iron oxide nanoparticles for interventional cardiovascular magnetic resonance imaging. Biomed. Microdevices.

[181] Liu H., Jiang Q., Huo J., Zhang Y., Yang W., Li X. (2020). Crystallization in additive manufacturing of metallic glasses: A review. Addit. Manuf..

[182] Lee Y., Lee C.H. (2018). Augmented reality for personalized nanomedicines. Biotechnol. Adv..

[183] Salavitabar A., Armstrong A.K. (2020). Personalized Interventions: A Reality in the Next 20 Years or Pie in the Sky. Pediatr. Cardiol..

[184] Lee H., Tajmir S., Lee J., Zissen M., Yeshiwas B.A., Alkasab T.K., Choy G., Do S. (2017). Fully Automated Deep Learning System for Bone Age Assessment. J. Digit. Imaging.

[185] Balu A., Nallagonda S., Xu F., Krishnamurthy A., Hsu M.-C., Sarkar S. (2019). A Deep Learning Framework for Design and Analysis of Surgical Bioprosthetic Heart Valves. Sci. Rep..

